# Interpersonal sensitivity on college freshmen’s depression: A moderated moderation model of psychological capital and family support

**DOI:** 10.3389/fpsyt.2022.921045

**Published:** 2022-07-29

**Authors:** Haibo Xu, Lixin Peng, Zhen Wang, Ping Zeng, Xin Liu

**Affiliations:** ^1^Center for Mental Health Education and Research, Xuzhou Medical University, Xuzhou, China; ^2^School of Management, Xuzhou Medical University, Xuzhou, China; ^3^Department of Epidemiology and Biostatistics, School of Public Health, Xuzhou Medical University, Xuzhou, China

**Keywords:** interpersonal sensitivity, depression, psychological capital, family support, moderated moderation model, freshman

## Abstract

**Background:**

The prevalence of depressive symptoms has become very high among college freshmen, with interpersonal sensitivity serving as an important predictor of depression. Combining internal and external positive resources can effectively prevent and alleviate depression. This study explores the moderating role of psychological capital (PsyCap) in the relationship between interpersonal sensitivity and depression, as well as the moderating effect of familial support on the conditional influence of PsyCap among Chinese college freshmen.

**Methods:**

A cross-sectional mental health survey was performed and the anonymous self-reported questionnaires, including the Patient Health Questionnaire, interpersonal sensitivity subscale of Symptom Checklist-90, Psychological Capital Questionnaire 24, and Perceived Social Support from Family, were distributed to the freshmen. Pearson’s coefficient was employed to describe correlations between variables. The PROCESS macro and slope difference tests were used to explore the moderating role of PsyCap and family support in the relationship between interpersonal sensitivity and depression.

**Results:**

The prevalence of depression among freshmen was 30.89% (694/2,247). The correlation analysis revealed that depression negatively related to PsyCap (*r* = −0.187, *p* < 0.001) and family support (*r* = −0.193, *p* < 0.001) and positively related to interpersonal sensitivity (*r* = 0.399, *p* < 0.001). The moderation analysis showed that PsyCap negatively moderated the positive relationship between interpersonal sensitivity and depression (β = −0.159, *p* < 0.001). We also found that family support played a moderating role in the conditional influence of PsyCap (β = 0.076, *p* < 0.01). The slope difference test further showed that family support weakened the effect of interpersonal sensitivity on depression in freshmen when they had low PsyCap.

**Conclusion:**

More attention should be paid to freshmen’s mental health and interpersonal interaction problems. For freshmen with interpersonal sensitivity and depression, mental health departments can conduct PsyCap development interventions to alleviate psychological symptoms. Freshmen themselves should also seek family support in time, but those individuals with high PsyCap should seek an appropriate level of family support to maintain their autonomy.

## Introduction

Depression is a common worldwide psychiatric disorder characterized by sadness, loss of interest or pleasure, feelings of tiredness, and poor concentration (1). Depression can reduce an individual’s work efficiency and job performance (2), increase the burden of healthcare (3), and even cause suicidal behavior in extreme cases (4). It has been well established that interpersonal problems act as risk factors for depression. In particular, several studies have indicated that poor quality of interpersonal relationships can predict individuals’ depressive symptoms (5, 6). According to the need to belong theory, humans have an intrinsic need for social connection. When the need to belong theory remains unmet, the individual’s behavior, cognition, and physical and mental health can be damaged (7). Impaired interpersonal relationships are a typical manifestation of this unmet need, and depression is one of the main consequences of interpersonal trauma (8). Previous work has argued that interpersonal relationships are integral to human wellbeing (9); we, therefore, need to manage interpersonal relationships well in daily life. However, individuals sometimes pay too much attention to their own relationships and fear the rejection or criticism of others in social interactions, which are symptoms of interpersonal sensitivity. Interpersonal sensitivity represents a set of symptoms that is likely to lead to the development of depression (10, 11). Researchers have attributed it to a personality trait in follow-up studies (12). Individuals with interpersonal sensitivity are extremely sensitive to the feelings of others and any feelings of discomfort during interpersonal interactions. Their sensitivity to others’ perceptions, especially in the form of rejection and criticism, leads them to modify their behavior so as to experience less rejection and criticism (10, 12). Previous research has indicated that interpersonal sensitivity can predict depression. Those with interpersonal sensitivity often have negative self-cognition due to their feelings of personal inferiority in comparison to other persons (13), and negative cognitions have long been considered central to depression (14). In addition, individuals with a high level of interpersonal sensitivity tend to suffer from interpersonal stress in social interactions, which serves as the most significant predictor of depression (15).

College students are at high risk of depression, and freshmen tend to experience higher levels of depression than non-freshmen (16). Within Erik Erikson’s theory of the eight stages of psychosocial development, traditional-age freshmen (18 years) would be in the fifth stage of development, which involves confronting the problem of identity and role confusion. This stage is vital for the personality development of freshmen (17). A study of nursing students indicated that 61.7% of first-year and 38.1% of last-year college students suffered from various degrees of depressive symptoms (18). Results of the meta-analysis about the prevalence of depression among college students revealed that 33.6% of students reported depressive symptoms (19), and the prevalence of depression among freshmen was 35.4% in Tang’s study (20). According to the report on the Development of China’s National Mental Health (2019–2020), the detection rate of depression among Chinese adolescents is 24.6%, and for major depression, 7.4%. According to a mental health survey of 1,048 freshmen in China, the detection rate of depression is about 65.6% (16). This recent research discovered that a total of 16.2% of freshmen (1,488 persons) exhibited positive results for depression among 9,013 Chinese samples that were screened by the SCL-90 (21). Depression and interpersonal sensitivity factors were the main characteristics examined. The first year of college serves as an important period of growth for adolescents, as college freshmen must learn to deal with managing their own academic studies, build relationships with new peers and teachers, and cope with potential financial problems (22). If they do not deal with these challenges in an appropriate way, they may develop mental health problems, such as anxiety and depression. Among freshmen, psychological problems most commonly stem from difficulties in interpersonal relationships (23, 24), including relationships with parents, teachers, peers, and romantic partners. Being away from parents may present a challenge to those who have not cultivated the ability to live independently. Research has discovered that a high quality of peer relationships benefits the mental health of adolescents (25). The study among Chinese college students indicated that interpersonal sensitivity positively relates to negative emotions (26) and contributes to individuals’ mobile phone addictions (27), which could predict depression (28). Nonetheless, only a few explorations about the relationship between interpersonal sensitivity and depression among Chinese college freshmen have been conducted.

Although group sandplay can reduce a person’s level of interpersonal sensitivity (29), the internal resources of individuals should also be developed to alleviate interpersonal sensitivity and depression. With the rise of positive psychology, many studies began to explore individuals’ positive psychological qualities and resources. Positive psychology advocates for using scientific methods to study and develop individuals’ positive resources to improve personal wellbeing. Seligman articulated three pillars of positive psychology: positive experience, positive personality, and a positive social organization system (30). In 2004, Luthans proposed the concept of psychological capital (PsyCap), defined as a positive psychological state expressed by individuals in the process of growth and development (31). PsyCap consists of four components: self-efficacy, optimism, hope, and resilience (32). Previous studies have discovered that PsyCap is negatively correlated with depression and could alleviate depressive symptoms (33–35). Furthermore, PsyCap was demonstrated to have a positive moderating effect on the relationship between work–family conflict and depressive symptoms in Chinese nurses (36). In addition, PsyCap can moderate the relationship between perceived stress and negative emotions (37), as well as the influence of interpersonal adaptation on internet addiction among college students (38). However, the moderating role of PsyCap in the relationship between interpersonal sensitivity and depression among Chinese college freshmen remains unclear. Thus, we selected PsyCap as the indicator of positive personality in the present study.

The moderating role of PsyCap as an internal factor in depression needs to be explored, while the role of external factors cannot be ignored. Many studies have shown that a combination of internal and external factors has a significant effect on the regulation of the poor conduct of adolescents (39), mitigating distress among persons who experienced stressful medical events (40), and improving students’ quality of life (41). Social support, as a common external resource, has been proven a crucial social factor that benefits human health (42). As a stress buffer, social support can alleviate physical and mental health (43). Further, as an important aspect of a positive social organization system, in Chinese traditional culture, the importance of the family in individual development cannot be ignored (44). Family support—a major part of social support—could be defined as the material, informational, and emotional support that an individual receives from family members (45, 46). Previous research has discovered an association between a low level of family support and adolescent depression (47). Family support was demonstrated to have the potential to ease depressive symptoms among college students (45). Moreover, family support can significantly alleviate depression in college freshmen when they have a low level of perceived stress reactivity (48). Although research has shown that parental rearing styles can predict different magnitudes of interpersonal sensitivity at varying levels (49), the relationship between family support and interpersonal sensitivity remains unclear.

**FIGURE 1:**
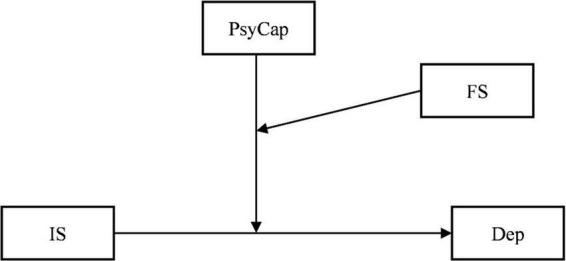
The moderated moderation model. In the figure, the independent variable is interpersonal sensitivity (IS), the dependent variable is depression (Dep), and the moderators are psychological capital (PsyCap) and family support (FS).

In conclusion, a wealth of literature has confirmed that interpersonal sensitivity can predict depression among college students. PsyCap and family support could alleviate depression. However, little research has studied the moderating effect of PsyCap on the relationship between interpersonal sensitivity and depression. Moreover, the functional efficiency of an individual’s internal resources is affected by external resources (50), and at the freshmen stage, the influence of family on psychological development would be more indirect in contrast to previous stages (17). Whether the psychological capital of college freshmen is affected by their family support remains unclear. Thus, our study further explores the moderating role of family support in the moderating effect of PsyCap and proposes several hypotheses, listed below, along with a hypothetical model (Figure 1):

**H1:** Interpersonal sensitivity has a significant correlative effect on depression among Chinese college freshmen.

**H2:** PsyCap can negatively moderate the relationship between interpersonal sensitivity and depression.

**H3:** Family support can moderate the conditional influence of PsyCap on the relationship between interpersonal sensitivity and depression.

## Materials and methods

### Subjects and sample selection

The mental health measurement of college freshmen, like physical examination, must be carried out routinely, supported by educational department policy, to ensure the healthy development of college students. In this study, a cross-sectional survey of freshmen was conducted at the end of September 2020 at a university in eastern China, with an anonymous self-reported questionnaire distributed to the freshmen. The purpose of the questionnaire and instructions for completing it were given to them by staff from the school organization departments.

The study was based on a mental health screening survey, conducted according to the guidelines of the Declaration of Helsinki, and approved by the ethical committee at the authors’ institution. The data collection process removed freshmen’s private information. Exclusion criteria for data included: (1) answers to questionnaire questions were incomplete, (2) answers were too short, and (3) random answers to questions or question choices had a clear pattern. For data that met the above conditions, we deleted the respondent’s whole set of answers. Since the survey was used to understand the mental health of freshmen, the study did not involve control variables other than gender.

The data acquisition process occurred as follows: the authors first obtained the authorization of relevant departments and the original data from 2,359 freshmen. Then, 112 pieces of data were deleted according to the exclusion criteria (the percentage of problematic data is 4.75%). Finally, the data used in this study were selected according to the threshold of the PHQ-9 (more than or equal to 5), producing a total of 694 samples.

### Measure

#### Depression

The Patient Health Questionnaire nine-item depression scale (PHQ-9) was used in this research. As the most widely used depression measure in the world, the PHQ-9 has shown strong reliability and validity in the study of population in different countries (51). It consists of nine items, with each item rated from 0 (“never”) to 3 (“almost every day”), and the possible total scores of 5, 10, 15, or 20 represent thresholds for mild, moderate, moderately severe, and severe depressive symptoms, respectively. In this study, the Cronbach’s α for the scale was 0.70.

#### Interpersonal sensitivity

The interpersonal sensitivity subscale of symptom checklist 90 (SCL-90) was employed (10). This instrument has been previously used with Chinese college students and has shown strong reliability (29, 52). The subscale includes nine items, and participants rated each item from 0 (“no”) to 4 (“very severe”). The sum of the scores from all items produces the interpersonal sensitivity score. A higher score indicates more problems in interpersonal interaction. In this study, the Cronbach’s α for the subscale was 0.85.

#### Psychological capital

The psychological capital of freshmen was measured *via* the Psychological Capital Questionnaire (PCQ-24) developed by Luthans (32). The PCQ-24 consists of four dimensions, with each dimension including six items. Each of the items is scored on a Likert six-point scale, with 1 indicating potent disagreement and 6 indicating strong agreement. A higher score generally indicates a higher level of PsyCap. In this study, the Cronbach’s α for PCQ-24 was 0.90.

#### Family support

A scale measuring perceived social support from family was adopted to measure individuals’ familial support. The scale was developed by Procidano (53) and included 20 items, each of which was rated 0 (“no”) or 1 (“yes”). The total possible score ranges from 0 to 20, with a higher score representing a higher level of perceived family support. In this study, the Cronbach’s α was 0.88. Since this instrument uses a 0–1-point scale, we further reported the KR-21 coefficient (54). The KR-21 coefficient of the scale for our study was 0.86.

### Statistical analysis

Harman’s single-factor test was conducted to test for common methods bias. Descriptive statistics (mean and standard deviation) and Pearson’s coefficient correlation were computed first. Then, an independent samples *t*-test was employed, followed by the moderation analysis using PROCESS macro ver3.5 developed by Hayes (55). We used Models 1 and 3 to explore the moderating role of PsyCap and familial support, respectively. All variables were standardized. In addition, we followed Dawson and Richter’s procedure (56) in testing for slope differences for the significant three-way interaction (interpersonal sensitivity, PsyCap, and familial support). SPSS 22.0 was employed to complete the above analyses, and all significance tests were two-sided. Furthermore, we adopted R 4.2.1 to conduct the power analysis of moderated tests.

## Results

### Preliminary analysis

Harman’s single-factor test analysis indicated that the variance explained by the first factor was 17.46%, which was less than the threshold of 40%. Therefore, the common method bias did not appear serious in the current study.

In our study, the prevalence of depression was 30.89% (694/2,247). Specifically, 591 freshmen exhibited mild depressive symptoms, 70 displayed moderate symptoms, 23 exhibited moderately severe symptoms, and 10 displayed severe depressive symptoms, according to the thresholds outlined in the method. To explore the influence of the underlying mechanism of PsyCap and familial support on depression, 694 freshmen with a sum score of depression ≥ 5 were selected. While 40.35% of these participants were men and 59.65% were women. The result of an independent samples *t*-test did not discover any difference in gender between all variables (*p* > 0.05).

### Correlation analysis

Mean, standard deviation, and correlation coefficients of all variables are shown in Table 1. The Pearson’s correlation analysis indicated that depression negatively related to PsyCap and family support (*r* = −0.187 and −0.193, *p* < 0.001, respectively) and positively related to interpersonal sensitivity (*r* = 0.399, *p* < 0.001). In addition, a positive relationship between PsyCap and family support was identified (*r* = 0.229, *p* < 0.001).

**TABLE 1:** Descriptive statistics and correlation coefficients.

	M	SD	1	2	3	4
1. Dep	7.909	3.147	–			
2. IS	14.442	6.148	0.399***	–		
3. PsyCap	102.107	13.064	−0.187***	−0.361***	–	
4. FS	13.895	4.833	−0.193***	−0.286***	0.229***	–

M, mean; SD, standard deviation; Dep, depression; IS, interpersonal sensitivity; PsyCap, psychological capital; FS, family support. ***p < 0.001.

### Moderation analysis

First, we explored the moderating role of PsyCap in the relationship between interpersonal sensitivity and depression. As shown in Table 2, the results of Model 1 supported H1 and revealed that depression was positively affected by interpersonal sensitivity (β = 0.376, *p* < 0.001). The moderation analysis indicated that PsyCap played the role of negative moderation (β = −0.159, *p* < 0.001) in the relationship between interpersonal sensitivity and depression, supporting H2. Then, we examined how family support moderates the conditional influence of PsyCap. The results of Model 3 revealed that the interaction term of interpersonal sensitivity, PsyCap, and family support was significant (β = 0.076, *p* < 0.01; see Table 3).

**TABLE 2:** Model characteristics for moderation analysis (Y = depression).

Variables	Coefficient	SE	*t*	LLCI	ULCI
Constant	−0.057	0.036	−1.598	−0.128	0.013
IS(X)	0.376	0.037	10.248***	0.304	0.448
PsyCap(M)	−0.050	0.037	−1.372	−0.122	0.022
X × M	−0.159	0.031	−5.190***	−0.219	−0.100
*R* ^2^	0.193				
*F*	54.944***				

Y, dependent variable; X, independent variable; M, moderation variable; IS, interpersonal sensitivity; PsyCap, psychological capital; SE, standard error; LLCI, lower level of the 95% confidence interval; ULCI, upper level of the 95% confidence interval. ***p < 0.001.

**TABLE 3:** Model characteristics for moderated moderation analysis (Y = depression).

Variables	Coefficient	SE	*t*	LLCI	ULCI
Constant	−0.058	0.036	−1.584	−0.129	0.014
IS(X)	0.343	0.038	9.051***	0.269	0.418
PsyCap(M)	−0.006	0.039	−0.164	−0.083	0.070
X × M	−0.106	0.036	−2.971**	−0.176	−0.036
FS(W)	−0.033	0.038	−0.871	−0.106	0.041
X × W	−0.032	0.035	−0.902	−0.101	0.038
M × W	0.011	0.035	0.314	−0.058	0.080
X × M × W	0.076	0.027	2.802**	0.023	0.130
*R* ^2^	0.207				
*F*	25.6511***				

Y, dependent variable; X, independent variable; M, the first moderation variable; W, the second moderation variable; IS, interpersonal sensitivity; PsyCap, psychological capital; FS, family support; SE, standard error; LLCI, lower level of the 95% confidence interval; ULCI, upper level of the 95% confidence interval. **p < 0.01, ***p < 0.001.

**FIGURE 2:**
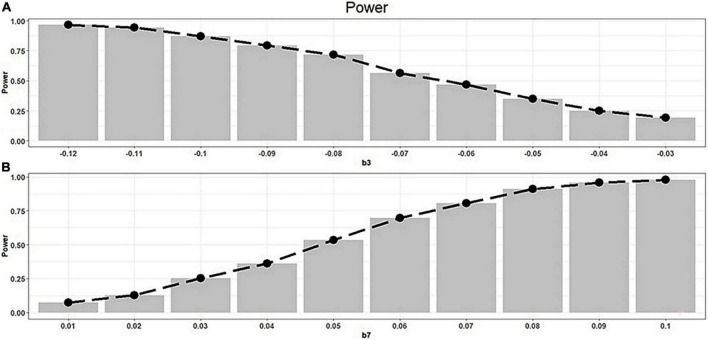
The average power for moderated moderation model. **(A)** Average power for testing whether *b*_3_ = 0. **(B)** Average power for testing whether *b*_7_ = 0.

We performed a power assessment following the moderation model obtained in our analysis:

$$\text{Y}=-0.058+0.343\,X-0.006\,M-b_{3}\,\mathrm{XM}-0.033\,W-\text{0.032XW+0.011MW}+b_{7}\,\mathrm{XMW}+\varepsilon ,$$

setting *b*_3_ = −0.03, −0.04, −0.05, −0.06, −0.07, −0.08, −0.09, −0.1, −0.11, or −0.12; *b*_7_ = 0.076 for scenario (A); and *b*_7_ = 0.01, 0.02, 0.03, 0.04, 0.05, 0.06, 0.07, 0.08, 0.09, or 0.1; and *b*_3_ = −0.106 for scenario (B). For all scenarios, the noise term εwas available from a normal distribution with mean 0 and SD 0.8. We tested whether *b*_3_ = 0 for scenario (A) and whether *b*_7_ = 0 for scenario (B), respectively. We repeated the experiment 1,000 times and estimated the power, which was defined as the proportion of truly detecting a non-zero effect size at the significance level of 0.05. More specifically, we gained a power of 90.8% when *b*_3_ = −0.106 and gained a power of 87.7% when *b*_7_ = 0.076, demonstrating that our moderated moderation model effectively detected moderation effect size and moderated moderation effect size (see Figure 2).

**FIGURE 3:**
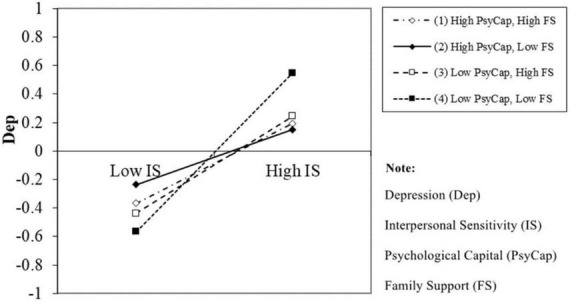
Three-way interaction plot.

Following Dawson and Richter’s procedure, we probed the relationship between PsyCap and interpersonal sensitivity and depression for each subgroup of family support (low and high), separately. Figure 3 indicates that the effect of interpersonal sensitivity on depression was lowest for freshmen with high PsyCap and a low familial support level.

Among the six pairs of slopes, we found three significant slope differences (Table 4). The first demonstrated that the slope with high PsyCap and family support was lower than that with low PsyCap and family support (*b* = −0.276, *p* = 0.003). The second significant slope difference was between freshmen with high and low PsyCap at a low level of familial support (*b* = −0.364, *p* < 0.001). The last indicated a significant slope difference in the interpersonal sensitivity-depression link between high and low family support for low PsyCap freshmen (*b* = −0.216, *p* = 0.014). In summary, familial support strengthened the attenuating effect of PsyCap on interpersonal sensitivity. Hence, this analysis supported H3.

**TABLE 4:** Slope difference test.

Pair of slopes	Slope difference	z	LLCI	ULCI
(1) and (2)	0.088	0.969	−0.090	0.266
(1) and (3)	−0.060	−0.583	−0.262	0.142
(1) and (4)	−0.276	−2.936**	−0.460	−0.092
(2) and (3)	−0.148	−1.391	−0.357	0.061
(2) and (4)	−0.364	−4.915***	−0.509	−0.219
(3) and (4)	−0.216	−2.472*	−0.387	−0.045

Slope 1: high PsyCap and family support; slope 2: high PsyCap and low family support; slope 3: low PsyCap and high family support; slope 4: low PsyCap and family support. LLCI, lower level of the 95% confidence interval; ULCI, upper level of the 95% confidence interval. *p < 0.05, **p < 0.01, ***p < 0.001.

## Discussion

This study adopted an interactionist approach exploring the use of internal and external resources to mitigate depressive symptoms caused by interpersonal sensitivity *via* investigating the role of Chinese college freshmen’s PsyCap (internal resource) and familial support (external resource). First, we tested a moderation model wherein the path from interpersonal sensitivity to depression varied at different levels of PsyCap. Second, we explored a moderated moderation model to evaluate the moderating role of family support in the conditional influence of PsyCap on the relationship between interpersonal sensitivity and depressive symptoms.

In our study, the prevalence of depressive symptoms was 30.89%, with no significant difference in the score of depression in terms of gender. For freshmen of different genders who have just entered the university, it may take some time to adapt to the strange environment and the challenges of interpersonal relationships, the results imply.

The results of the moderation model supported H1, consistent with previous research (13, 57). Compared with life in high school, university life entails more free time for freshmen to build their private lives, but challenges follow. In high school, most students come from the same area, and some may even be neighbors, sharing the same regional culture, such as eating habits and speaking style (e.g., dialect). In contrast, freshmen face a strange living environment and new classmates from different districts. According to the conservation of resources theory (COR) (58), the loss of individual resources can cause a stress response, such as depression. The resource investment principle indicates that investing in existing resources can prevent future resource losses. Therefore, freshmen must build strong interpersonal relationships, which will help provide a strong foundation for future study and life. However, failure in social interactions can result in cumulative interpersonal relationship risks that can decrease an individual’s resilience (23). In addition, maladaptation to the surrounding environment also hinders the development of good interpersonal relationships (59). Due to their introverted or shy character, some freshmen became too cautious and develop a sense of inferiority within interpersonal interactions—a typical symptom of interpersonal sensitivity (10). If the intervention does not happen in time, it will develop into depression (60). Timely intervention can effectively prevent and alleviate the interpersonal sensitivity and depressive symptoms of freshmen.

The findings from this study highlight that PsyCap plays a moderating role in the process of the mitigation of interpersonal sensitivity to depression. Compared to those with low PsyCap, freshmen with high PsyCap show fewer depressive symptoms caused by interpersonal sensitivity. As a comprehensive complex of multiple positive traits, PsyCap has led to significant reductions in depressive symptoms among patients (61). Impaired interpersonal relationships can lead to unmet needs for belonging (7), decrease an individual’s resilience (23), and develop into depression. According to the gain paradox principle, resources gain more importance when individuals lose some resources (58), meaning individuals fear losing the remaining resources even more. One reason for studying psychological capital is that it can be explored and developed. Previous research has discovered that optimism can moderate the effect of thwarted belongingness on suicidal ideation (62), and self-efficacy was found to contribute to interpersonal behavior (63). After failing in interpersonal interaction, freshmen with high self-efficacy and optimism are not afraid of interacting with others again. Research has shown that school adaptation can be indirectly affected by interpersonal relationships *via* resilience among Chinese university students (64), and military training can reduce depression in freshmen by improving psychological resilience (65). In addition, hope serves as a predictor of negative affective conditions linked to interpersonal violence among students in China (66). As a high-order positive resource, PsyCap offers stronger psychological protection than any other single resource. A high level of PsyCap can replenish the resources consumed in interpersonal failure and further reduce depression in freshmen. Intervention should be conducted with those freshmen with low PsyCap to prevent and reduce their depressive symptoms. In addition, the direct moderating effect of familial support did not appear significant in our study. We inferred that family support directly moderates the relationship between interpersonal sensitivity and depression only when it is sensed and utilized by individuals. According to Beck’s cognitive theory, depressed individuals have cognitive biases that cause them to tend to ignore positive information and pay more attention to negative information (67). Therefore, depressed freshmen are unlikely to take initiative to receive family support, which prevents the family support from moderating the relationship between interpersonal sensitivity and depression.

The present study has discovered that family support has a moderating effect on the conditional influence of PsyCap. Specifically, in the context of low PsyCap, it was found that freshmen with high levels of family support exhibit less depression than those with low family support. When freshmen have insufficient development of internal resources, the role of external resources becomes more critical. Previous research has indicated that increasing the familial support of freshmen in the transitional stage of university may help to prevent depressive symptoms (48). Correct and appropriate family support has a lasting effect on individuals’ mental health because it shapes individuals’ internalized views of interpersonal relationships and their general expectations of whether they will be accepted or rejected by others (49). In addition, our study further reveals that freshmen with a high level of PsyCap and family support have a lower depression score than those with a low level of PsyCap and family support, and a slope difference test was significant. This result was predictable. Compared with those who lack resources, freshmen with high internal and external resources will certainly deal with depression more successfully. However, this result verified the corollary of the initial resource effect of COR. What is more is that the present study also found that freshmen with low PsyCap display more depression than those with high PsyCap in the context of low family support, which confirmed H2. Interestingly, although the slope difference was not significant, the results shown in Figure 3 reveal that freshmen with a lower level of family support have a lower depression score than those with high family support in the context of high PsyCap and high interpersonal sensitivity. We inferred that overly high family support destroys freshmen’s autonomy, especially in those with high PsyCap. Those with high PsyCap have a certain degree of confidence and ability to deal with their own problems. In line with the self-determination theory, overly high family support can destroy their competence (propensity to be secure and confident in their own abilities) and autonomy (ability to make personal choices) (68, 69). Previous research has indicated that parental overprotection increases individuals’ interpersonal sensitivity (70). Their excessive involvement may reduce individuals’ autonomy and increase their fear of social life and difficulty in dealing with social relationships (71). In Chinese culture, it is true that some parents are overly involved in their children’s lives and want to arrange and plan everything for their children. For freshmen who have just entered college and are separated from their parents, this may facilitate some practical elements of their life, but it will also make them more prone to negative emotions in the case of interpersonal failure. Hence, for individuals with high internal positive resources, family members should provide appropriate familial support so as to cultivate their autonomy. The appropriate level of support is relative; however, for those with low PsyCap, very high family support would be the only positive support. They do not have enough self-ability to cope with depression, so they can only rely on attachment and support from their family. Thus, a very high level of family support would not disturb the autonomy of freshmen with low PsyCap.

This study has some limitations that must be acknowledged. First, due to its cross-sectional observational nature, our study was not effective enough to explain causality. Future studies should use longitudinal data to test moderating effects. Second, because the main purpose of the present study was to explore an intervention mechanism, it did not study the effect of covariates on depression, which may lead to bias in the outcomes. Future research should include appropriate control variables. Third, we have not explored the specific role of the components of PsyCap in the moderated mechanism. Fourth, there may be a bidirectional relationship between interpersonal sensitivity and depression (72, 73). Our study did not explore the effect of depression on freshmen’s interpersonal sensitivity, which requires further research. Fifth, the sample selection is relatively simple, all freshmen from a university in eastern China, which will affect the generalizability of our results.

## Conclusion

Our study revealed that PsyCap can negatively moderate the relationship between interpersonal sensitivity and depression among Chinese college freshmen. Moreover, family support can further weaken the effect of interpersonal sensitivity on depression among those with low PsyCap. To combat the issue of depression among college freshmen, school authorities should pay more attention to the mental health of freshmen. Mental health departments can carry out a psychological capital development intervention plan for those freshmen with depressive tendencies. Meanwhile, the families of freshmen should provide appropriate familial support to help the freshmen through the transition period.

## Data availability statement

The raw data supporting the conclusions of this article will be made available by the authors, without undue reservation.

## Author contributions

HX contributed to the conception and design of the study, the manuscript preparation, and the final revision. LP performed the data analysis and drafted the initial manuscript. ZW revised the manuscripts. PZ guided the statistical analysis process. XL contributed to the conception and design of the study, investigated the data, and established the databases. All authors contributed to the manuscript, read, and approved the final version of the manuscript for submission.
